# The Role of Accumbal Hypoactivity in Cocaine Addiction.

**DOI:** 10.1100/tsw.2007.266

**Published:** 2007-11-02

**Authors:** L. L. Peoples, A. V. Kravitz, K. Guillem

**Affiliations:** ^1^ Department of Psychiatry, University of Pennsylvania School of Medicine, Philadelphia, PA 19104, USA; ^2^ Neuroscience Graduate Group, University of Pennsylvania School of Medicine, Philadelphia, PA 19104, USA

**Keywords:** plasticity, compulsive behavior, addiction, dopamine, psychomotor stimulants, addictive drugs

## Abstract

Cocaine-induced hypoactivity of the nucleus accumbens (NAC) is hypothesized to contribute to cocaine addiction. There are two important questions related to this hypothesis. First, cocaine addiction is characterized by an increase in drug-directed behavior and a simultaneous weakening of other motivated behaviors. However, the NAC contributes to both drug- and nondrug-directed behavior. Moreover, the nature of the contributions is similar and associated predominantly with excitatory phasic firing patterns. Given these observations it is not clear how hypoactivity of NAC neurons might contribute to the behaviors that characterize cocaine addiction. Second, various types of investigations have documented neurochemical and molecular adaptations that could underlie NAC hypoactivity. However, there is also evidence of other adaptations in the NAC, and in NAC afferents, which are expected to have an excitatory influence on NAC neural activity. In the present review we will briefly overview these issues. We will also describe a hypothesis, and related empirical evidence, that may contribute to answering these questions. Further investigation of the issues and the hypothesis may contribute to a better understanding of the neuroadaptations that contribute to cocaine addiction.

## THE ROLE OF ACCUMBAL HYPOACTIVITY IN COCAINE ADDICTION

Hypoactivity of the nucleus accumbens (NAC) and interconnected structures is hypothesized to contribute to cocaine addiction. The hypothesis is based on a number of basic and clinical findings. However, there are also observations that are difficult to reconcile with this proposal. Herein, we will overview these various lines of evidence and describe a hypothesis and related empirical evidence that may provide answers to questions regarding the role of NAC hypoactivity in cocaine addiction.

## REPEATED COCAINE EXPOSURE IS ASSOCIATED WITH NAC HYPOACTIVITY

Multiple electrophysiological studies have demonstrated that repeated cocaine exposure can decrease NAC activity. Recordings conducted in anesthetized animals have shown that the responsiveness of NAC

neurons to excitatory stimulation is reduced in animals with a history of experimenter-delivered cocaine[1,2]. Experiments in NAC slices have shown that excitatory currents and whole cell excitability are decreased in rats with a history of cocaine exposure[3,4]. Moreover, repeated injections of cocaine can lead to a decrease in the ratio of AMPA to NMDA currents (AMPA:NMDA currents), a mechanism linked to long-term-depression (LTD)[6, for related observations see 3,5,7]. Extracellular recordings in behaving animals have shown that basal firing rates of NAC neurons decrease across daily cocaine self-administration sessions[8,9]. The decreased basal activity occurs in association with escalations in cocaine self-administration. Taken as a whole, these data demonstrate that a history of repeated cocaine can be associated with decreases in excitatory synaptic and neuronal activity in the NAC. Moreover, the electrophysiological data are consistent with the hypothesis that cocaine-induced NAC hypoactivity contributes to the development of cocaine addiction.

Consistent with the rat electrophysiological studies, neuroimaging studies in primates have shown that a history of cocaine self-administration can enhance evidence of hypoactivity in the NAC and other related structures during periods of cocaine exposure[10,11]. Neuroimaging studies of cocaine-addicted humans have also implicated hypoactivity in cocaine addiction. These studies demonstrated that basal activity in the striato-thalamo-cortical circuit of cocaine-addicted individuals is hypoactive relative to that of control subjects[13,14]. It is not known whether the diminished neural activity in cocaine-addicted humans reflects either a pre-existing condition or a chronic drug effect. Nevertheless, the animal and human neuroimaging studies are collectively consistent with the hypothesis that hypoactivity contributes to cocaine addiction and justify further investigation of the role of pharmacological mechanisms[3,12,14].

## QUESTIONS ABOUT NAC HYPOACTIVITY

### Evidence that Cocaine Induces Excitatory as Well as Inhibitory Neuroadaptations

Neurochemical and molecular studies show that repeated cocaine exposure induces multiple adaptations in the NAC. Some of these adaptations are expected to have an inhibitory effect on NAC neural activity; however, others are expected to have the opposite effect. For example, repeated exposure to cocaine can lead to decreased basal presynaptic and extracellular glutamate levels in the NAC[15,16,17,18,19], which might be expected to have an overall inhibitory effect on tonic activity of NAC neurons. However, repeated cocaine also increases drug-evoked glutamate release[20] and surface AMPA receptors on medium spiny neurons[21,22,23], which might be expected to be excitatory. A history of cocaine exposure can also be associated with changes in nonglutamatergic neurochemicals[24,25] and receptors[26,27], as well as plasticity in growth factors[28], signal transduction pathways, gene transcription factors, and cell morphology[29,30,31,32,33]. Certain of these adaptations are expected to be inhibitory[30,32], but others are expected to be excitatory[20,23,28,30,31,34].

There are multiple potential explanations of the evidence for both excitatory and inhibitory plasticity. One hypothesis is that repeated cocaine induces hypoactivity and that certain excitatory adaptations reflect a compensatory response that tends to oppose that hypoactivity[23,35,36]. Consistent with this proposal, extended abstinence is associated with numerous adaptations that are expected to have an excitatory effect on NAC neural activity[20,23,37,38]. Also consistent with the proposal are the findings of four recent electrophysiological investigations, which conducted novel recordings in animals exposed to extended cocaine abstinence. In two of the studies, Hollander and Carelli exposed animals to multiple daily cocaine self-administration sessions followed by either 1 or 30 days of abstinence[39,40]. These investigators observed that phasic firing patterns time locked to cocaine-directed operant behavior were increased in prevalence and magnitude at the late abstinence time-point relative to the early time point. In a third study, Kourrich et al. exposed animals to repeated experimenter-delivered cocaine, followed by either 1 or 10–14 days of abstinence[131]. Recordings conducted in NAC slices of these animals showed that at the early abstinence time point, the ratio of AMPA:NMDA currents was reduced in the cocaine-treated animals relative to that in nondrug controls. The opposite finding was observed at the late abstinence time point. In a fourth study, similar to that of Kourrich et al., Martin et al. observed less evidence of LTD-like adaptations after 21 days of abstinence relative to 1 day of abstinence[41,131]. All four of these electrophysiological studies differ from most other relevant studies in that recordings included late as well as early abstinence time points. Together, the available electrophysiological data, along with other observations, are consistent with the interpretation that repeated cocaine induces hypoactivity in the NAC, although this type of adaptation may be more prominent under conditions of limited drug abstinence.

The interpretation may represent a partial explanation for the evidence of both excitatory and inhibitory cocaine-induced adaptations. One might further speculate that inhibitory adaptations are more involved in the development of addiction than in the expression of the disorder. However, these proposals are not wholly consistent with certain observations. First, the Kourrich et al. study showed that the increased AMPA:NMDA current ratio that was associated with extended abstinence could be reversed if the long-abstinent animals were re-exposed to cocaine 24 h prior to the recording session[131]. Based on this observation, it is possible that some inhibitory adaptations contribute to both the development and expression of cocaine-addicted behavior. Second, neuroimaging studies of cocaine-addicted humans show evidence of persistent hypoactivity in striatal and cortical regions. It is possible that the persistent hypoactivity in humans is nonpharmacological in nature. Alternatively, cocaine-induced hypoactivity might become more persistent as drug exposure exceeds that which has been employed in most animal studies. Additional parametric studies are needed to characterize the role of hypoactivity and other adaptations in the development and expression of cocaine addiction.

### The Role of the NAC in Both Cocaine Addiction and Normal Motivated Behavior

The development of drug addiction is associated with both "positive" and "negative" symptoms (i.e., emergence of abnormal behavior and loss of normal function, respectively). The positive symptoms include compulsive and uncontrollable drug-directed behavior. Collectively, the negative symptoms can be characterized as reflecting a general disruption or weakening of normal motivated behavior. The symptoms can include decreased interest in alternative motivated behaviors, decreased time spent engaging in those behaviors, anhedonia, and a decreased ability to use information about negative behavioral outcomes to inhibit potentially disadvantageous and even harmful behavior[13,42,43,44].

The NAC and interconnected structures facilitate both drug- and nondrug-related motivated behaviors[45,46,47,48,49,50,51]. The functional contribution of the NAC to drug- and nondrug-related behaviors is similar[52,53,54,55]. Neuropharmacological evidence also shows that both drug- and nondrug-related instrumental behaviors are regulated by glutamatergic NAC mechanisms[19,56,57,58, 59,60,61]. Moreover, electrophysiological recordings in behaving animals have shown that the patterns of NAC neurons time locked to instrumental behavior are quite similar for drug and nondrug rewards, consisting predominantly of phasic increases in firing[56,62,63,64,65]. Given these observations, it is unclear how decreased synaptic and neuronal activity in the NAC could contribute to a selective increase in drug-directed behavior and a concomitant weakening of other motivated behaviors. Indeed, one might expect that NAC hypoactivity would contribute to a general weakening of all motivated behaviors.

## DIFFERENTIAL NEUROPLASTICITY

We have highlighted two basic questions regarding NAC hypoactivity. First, how might we reconcile evidence of NAC hypoactivity with evidence for excitatory as well as inhibitory adaptations in the NAC? Second, how might NAC hypoactivity contribute to an increase in drug-directed behavior and a weakening of other motivated behaviors given that the NAC role in those behaviors is similar and associated with excitatory NAC mechanisms? Addressing the questions is important to evaluating whether and how NAC hypoactivity contributes to drug addiction.

Observations of Berke and Hyman[69] and a hypothesis that we have proposed[66,67,68] may provide answers. In considering the role of striatal and cortical adaptations in drug addiction, Berke and Hyman noted that uniform neuroadaptations within the individual structures could not explain the selective change in drug-directed behavior that characterizes addiction[69]. The authors proposed that neurons that are responsive to drug-associated cues and contribute to drug-directed behavior are selectively activated during periods of drug self-administration. Because of the selective activation, these neurons are uniquely impacted by activity-dependent, excitatory adaptations, such as long-term potentiation (LTP), which typically underlie learning and which are induced by cocaine. The authors hypothesized that the unique adaptations could selectively enhance neuronal responses to drug-associated cues and contribute to selective increases in drug-directed behavior. We propose that "differential neuroplasticity" may contribute even more broadly to the pattern of neuroadaptations that underlie addiction.

In brief, we propose that synapses and neurons are differentially activated during periods of drug exposure: Some are excited, whereas others are quiescent or inhibited. These differences in activation make synapses and neurons differentially susceptible to multiple types of drug-induced plasticity. In some cases, the net effect of the differential adaptations is to establish chronic between-synapse and between-neuron differences in excitability and activity that are similar to those that are present during cocaine self-administration, and that selectively facilitate drug-directed behavior[66,67,68].

Differential neuroplasticity may occur in multiple components of the striato-thalamo-cortical circuits. However, we have thus far specifically considered the role of differential adaptations with respect to NAC hypoactivity (referred to as the differential inhibition hypothesis). The latter more specific hypothesis can be broken down into four main components, which are described herein.

1. A particular spatial distribution of synaptic and neuronal activity in the NAC promotes drug-directed behavior — Electrophysiological recordings conducted in behaving animals have demonstrated that NAC neurons exhibit heterogeneous patterns of activity during motivated behaviors. Some neurons are excited; whereas others are quiescent or even inhibited. These spatial patterns of activity appear to be unique to particular motivated behaviors. Based on these observations, we hypothesize that unique spatial distributions of activity across NAC synapses and neurons are associated with, and selectively facilitate, particular motivated behaviors.
2. Cocaine has activity-dependent acute effects on NAC activity — Available evidence shows that DA is a primary mediator of the acute pharmacological effects of cocaine on NAC neural activity. DA effects on NAC firing can be activity dependent. Based on the nature of those activity-dependent effects, we hypothesize that when drug-directed behavior leads to cocaine intake, the acute pharmacological actions of cocaine inhibit NAC neurons in an activity-dependent manner: Synapses and neurons receiving strong excitatory input at the time of drug exposure are less inhibited than are synapses and neurons receiving weak excitatory input. The activity-dependent acute effects of cocaine exaggerate differences in activity between the activated and nonactivated synapses and neurons.
3. Neuroadaptations that mediate cocaine-induced hypoactivity are activity dependent — We hypothesize that excitatory synaptic and neuronal activity opposes mechanisms that lead to hypoactivity. Thus, differences in afferent input and acute cocaine-induced inhibition during a period of cocaine self-administration make synapses and neurons differentially susceptible to mechanisms that mediate hypoactivity. Synapses and neurons that are activated during periods of drug self-administration are less susceptible to the mechanisms that mediate hypoactivity than are other, less activated, synapses and neurons. In addition, activated synapses and neurons may be more subject to excitatory plasticity mechanisms. The susceptibility differences lead to differential neuroadaptations, which maintain or enhance the activity of synapses and neurons that are strongly active during cocaine exposure and decrease activity of synapses and neurons that are less active during cocaine self-administration.

4. The differential neuroadaptations alter the basal spatial pattern of excitability and activity so that it is consistent with a pattern that selectively facilitates drug-directed behavior — The differential adaptations are hypothesized to alter the basal spatial pattern of excitability and activity so that it is abnormally and chronically more consistent with that which is present during periods of drug exposure. Additionally, the altered baseline spatial pattern might correspondingly increase the between-synapse and neuron differences in activity that normally occur in association with drug seeking and taking. The altered basal topography of excitability and activity is hypothesized to amplify, and to some degree "lock in", spatial patterns of activity that selectively promote drug-directed behavior. Concurrently, the altered spatial pattern of excitability and activity interferes with the transitions in synaptic and neuronal activity that are necessary for nondrug-related motivated behaviors to occur.

## POTENTIAL ANSWERS TO QUESTIONS ABOUT THE ROLE OF NAC HYPOACTIVITY IN COCAINE ADDICTION

### Evidence that Cocaine Induces Excitatory as Well as Inhibitory Neuroadaptations

Differential activity-dependent neuroplasticity is potentially relevant to reconciling evidence of both excitatory and inhibitory cocaine-induced neuroadaptations in the NAC. Specifically, it is possible that excitatory and inhibitory adaptations occur concurrently, but develop differentially among synapses and neurons that are differentially active during periods of cocaine exposure. It is possible that differential adaptations, once they have occurred, establish differential susceptibility to certain additional adaptations, such as the excitatory adaptations that have been observed to occur during abstinence. Additionally, between-synapse and neuron differences in basal excitability and activity, once established, could potentially persist even if there were shifts in the predominance of inhibitory vs. excitatory adaptations across different stages of the addiction cycle.

### The Role of the NAC in Both Cocaine Addiction and Normal Motivated Behavior

The differential inhibition hypothesis of NAC hypoactivity provides a potential explanation as to how "hypoactivity" in the NAC could contribute to the changes in drug- and nondrug directed behaviors that define cocaine addiction[66,67,68]. The proposed shift in the basal pattern of excitability and activity is hypothesized to be chronic. The altered pattern of excitability and activity is also proposed to be consistent with that which facilitates drug-directed behavior and, hence, to be inconsistent with that which is optimal for other motivated behaviors. If these proposals were true, the accumbens would be in a state that persistently biased individuals to engage in drug-directed behavior rather than other motivated behaviors. The differential neuroadaptations could contribute to the emergence of compulsive drug-directed behavior and a concomitant decline in other motivated behaviors.

The hypothesis also provides a closely related explanation of how hypoactivity might selectively enhance drug-directed behavior when that behavior, like other motivated behaviors, is associated with predominantly excitatory phasic activity. The proposed shift in basal excitability and activity is hypothesized to amplify differences in activity between synapses and neurons that do and do not receive excitatory input in association with drug-directed behavior. The increased difference would increase the "signal-to-background" ratio between the excitatory phasic signals associated with drug-directed behavior, and those associated with other motivated behaviors. The increased signal-to-background ratio could selectively increase the impact of the excitatory phasic firing patterns that facilitate drug-directed behavior. Such changes in phasic activity might be expected to enhance sensitivity of individuals to events that evoke drug-directed behavior, strengthen drug-directed behavior, and weaken other motivated behaviors. This proposal is consistent with mechanisms described by several other authors[14,70,71].

## BASIS OF THE DIFFERENTIAL INHIBITION HYPOTHESIS OF NAC HYPOACTIVITY

The differential inhibition hypothesis is based on three main areas of research, which have characterized the following: (1) firing patterns of NAC neurons during drug- and nondrug-related motivated behaviors, (2) effects of DA and cocaine on activity of NAC neurons, and (3) mechanisms that mediate NAC hypoactivity. These areas of research are overviewed herein.

### Firing Patterns of NAC Neurons during Drug- and Nondrug-Related Motivated Behavior

#### *Distinct Spatiotemporal Patterns of NAC Activity Facilitate Different Motivated Behaviors*

The connectivity of the NAC positions the structure to transmit limbic, motivationally relevant, information to circuits that contribute to decision-making and motor planning. It is proposed that this NAC contribution influences which limbic signals are most likely to determine behavior[66,68,72,73, 74,75,76,77,78,79,80,81,82]. Consistent with these views, it has been hypothesized that "… behaviorally meaningful information in the NAC is represented by fine-grained spatio-temporal firing patterns [of ensembles of neurons]… rather than by massive waves of activity uniformly sweeping from NAC to the ventral pallidum and related terminal fields"[83].

One hypothesis regarding the fine-grained spatial patterns of activity is that they reflect activity of distinct subsets of neurons that contribute to particular motivated behaviors. Alternatively, the spatial patterns of activity might reflect different patterns of activity exhibited by largely overlapping groups of neurons. There is evidence to support both proposals, although the latter appears more likely to be correct. Recordings conducted in behaving animals show that subsets of neurons exhibit phasic responses during appetitive, instrumental behavior[39,62,63,65,66,67,68,84,85,86,87]. Moreover, NAC neurons that show a phasic response time locked to cocaine-reinforced lever presses are largely distinct from those that show a similar firing pattern in relation to food or water-reinforced lever presses[88,89,90,91]. However, the neurons that show the firing patterns time locked to the cocaine- and food/water-reinforced operant together account for ~50% of all recorded neurons. This theoretically leaves only 50% of recorded neurons in the NAC to mediate all other motivated behaviors. Additionally, other studies that have examined phasic activity time locked to multiple discrete events within an appetitive sequence (e.g., lever presses, onset of reward consumption) have observed that many more accumbal neurons show a phasic firing pattern in relation to at least one event. This number increases if phasic activity time locked to particular events is characterized on different time bases[67,85]. Finally, if one considers tonic as well as phasic changes in firing, the proportion of "responsive" neurons accounts for almost all recorded neurons[87]. Together, these findings are consistent with the interpretation that individual neurons show responses in relation to multiple motivated behaviors. However, the nature of the responses vary so that the overall spatiotemporal patterns of accumbal activity that are associated with different behaviors are unique[83,84,90].

#### *Firing Patterns Associated with the Cocaine-Directed Operant Behavior and Cues that Evoke Cocaine-Directed Behavior*

##### Phasic Firing Patterns Associated with Cocaine-Reinforced Behavior during Self-Administration Sessions

Some of the first extracellular recordings conducted during cocaine self-administration sessions characterized the phasic firing patterns time locked to the operant behavior. In these sessions, rats were trained to self-administer cocaine on a fixed-ratio 1 (FR1) schedule of cocaine reinforcement. In most experiments, completion of the press was followed immediately by presentation of conditioned reinforcers (i.e., tone or light cue) that signal the onset of the drug infusion. These studies[62,92,93,94] showed that NAC neurons exhibit rapid phasic changes in firing time locked to cocaine-reinforced lever presses (referred to herein as lever-press firing patterns, Fig. 1). The lever-press firing patterns vary in time course, but tend to begin during the 1–3 sec before the press and to persist for several seconds after the press. The phasic lever-press firing patterns have been observed under a broad range of conditions, but are consistently reported as predominantly excitatory[39,62,67,92,94].

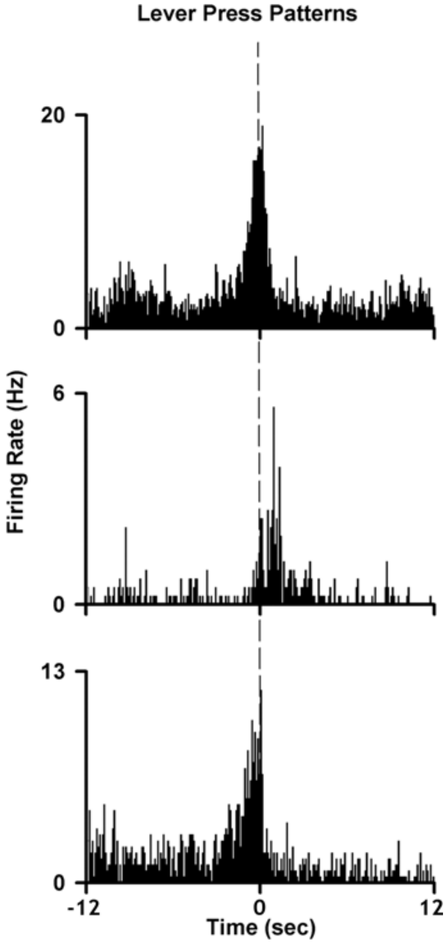

**FIGURE 1.** Phasic changes in firing time locked to the cocaine-reinforced lever press during a FR1 cocaine self-administration session. Each histogram shows an individual neuron example of an excitatory lever-press firing pattern. In each of the three histograms, average firing rate (Hz per 0.1-sec bin) is plotted during the 12 sec before and after presses made during the maintenance phase of the self-administration session (i.e., 10th – 27th reinforced lever presses). (Figure taken from Peoples et al (2004) *J. Neurophysiol.* **91,** 314–323. With permission.)

## Changes in Firing Associated with Presentation of Cues that Evoke Drug-Directed Behavior

Two published studies characterized NAC responses during cue-evoked drug-directed behavior[68,95]. In both studies, drug-free animals with a history of cocaine self-administration training were exposed to a cue that predicted the availability of cocaine. The response lever was available, but operant responses were not reinforced. The cue presentation evoked drug-directed instrumental behavior. A portion of NAC neurons showed a change in firing in association with the cue presentation (e.g., Fig. 2). Ensuing drug-directed instrumental behavior was also associated with changes in firing. In all cases, the changes in firing were predominantly excitatory (i.e., >85% of responsive neurons). Control analyses showed that the changes in firing were not attributable to motor behavior and did not reflect a primary sensory response.

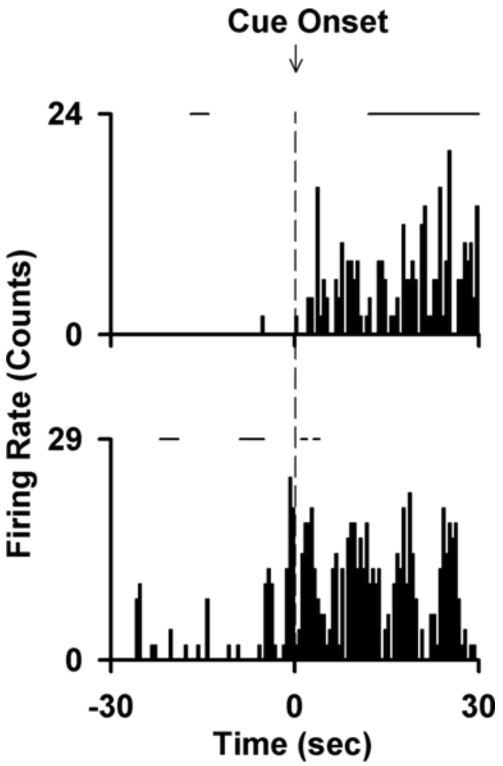

**FIGURE 2.** Examples of excitatory changes in firing time locked to presentation of cues that signaled the onset of a cocaine self-administration session. The change in firing time locked to the onset of a cue that signaled the start of a cocaine self-administration session is shown for each of two neurons (one per row). Firing rate (counts per 0.5-sec bin) is plotted for the 30-sec periods that preceded and followed onset of the cue. Cue onset is shown at Time 0 on the abscissa. Above each of the histograms are horizontal lines indicating periods of locomotion. Comparison of the timing of locomotion and the time course of the change in NAC firing shows that the latter is dissociated from, and thus not attributable to, the former. Firing patterns were observed in rats with a 2-week history of daily cocaine (0.75 mg/kg/inf) self-administration. The recording session consisted of a 20-min presession baseline period, a cocaine self-administration session, and a 40-min postsession baseline period. During the presession baseline and postsession recovery period, animals did not have access to the operant response lever and drug was not available. Animals initiated cocaine self-administration shortly after the onset of the cues, but not with less than a 1-min latency. (Figure taken from Peoples et al. (2004) *J. Neurophysiol.* 91, 314–323. With permission.)

In an additional study (unpublished), we recorded firing of NAC neurons during either a within-session cue reinstatement procedure or a within-session drug + cue reinstatement procedure. During these sessions, cocaine-reinforced behavior was first extinguished and then reinstated with presentation of either a compound cue that was typically associated with delivery of a cocaine reinforcer or the cue + a single infusion of cocaine. The response of NAC neurons to the cue and the cue + drug presentations were almost exclusively excitatory (see Figs. 3 and 4 for details). The evidence of predominantly excitatory changes in firing time locked to events that evoke drug-directed behavior is consistent with other evidence that excitatory NAC mechanisms contribute to cue- and drug-evoked drug seeking[96,97].

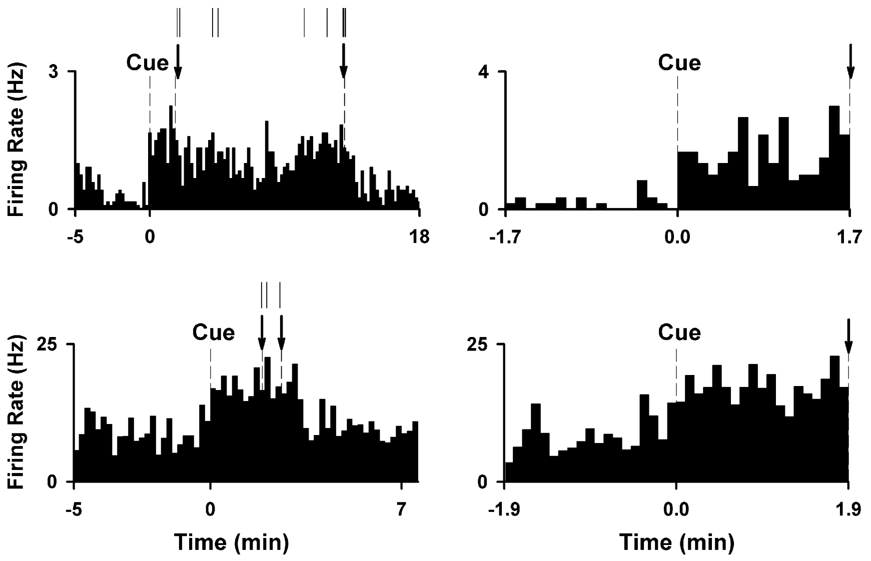

**FIGURE 3.** Examples of excitatory changes in firing during a within-session cue reinstatement procedure. Animals with a 30-day history of daily long-access (6 h, 0.75 mg/kg/inf) cocaine self-administration sessions were exposed to a within-session extinction and reinstatement procedure[135]. In brief, during the recording session, a regular self-administration session was followed by an extinction period in which operant responding was not reinforced. Animals first increased and then ceased responding during the extinction period. Thereafter, operant responding was reinstated by presentation of cues that were typically associated with delivery of cocaine infusions during the daily self-administration sessions. The average period of time that elapsed between the last drug infusion and presentation of the cue presentation equaled 86.6 ± 11.3 min. Average latency between onset of the cue and the first reinstated lever press was 2.5 min. Of the 40 recorded neurons, 18 showed a significant change in firing between the onset of the cue and the interval that elapsed before the first reinstated press. Except for one neuron, the change in firing rate was an increase. In the figure, firing before and after the onset of the cue are shown for each of two neurons (one neuron per row). (Left) Each histogram shows firing during pre- and postcue periods. The precue period is the 5 min that immediately preceded the onset of the cue. The postcue period is the interval that elapsed between the onset of the cue and the 5 min after the last reinstated press. The time base of the histogram is sufficiently long to display firing during the entire period of reinstated operant behavior, as well as during periods immediately before and after that period. Small vertical bars above each histogram correspond to lever presses; first and last reinstated presses are marked with arrows. (Right) Firing of the same neurons is shown during shorter pre- and postcue periods. The postcue period corresponds to the interval between the onset of the cue and the completion of the first reinstated press. The precue period is an interval of equal duration that occurred immediately before the onset of the cue.

## Relevance to the Differential Inhibition Hypothesis

As already noted, the characteristics of phasic firing patterns time locked to drug-associated cues and drug-directed operant behavior are consistent with patterns of NAC activity observed in studies of nondrug rewards. The recording studies are relevant to two main components of the differential inhibition hypothesis. First, it is proposed that neurons that exhibit excitatory phasic responses in association with drug-directed behavior are more active during cocaine self-administration than are neurons that normally exhibit excitatory firing patterns in association with nondrug-motivated behaviors. This difference in firing is proposed to establish conditions that lead to differential neuroadaptations. The differential plasticity weakens excitatory phasic firing patterns that normally occur in relation to cues and behavior associated with nondrug rewards. In contrast, the excitatory phasic firing patterns that normally occur in relation to drug-associated cues and behavior are potentially maintained or even enhanced. Second, these differential changes in phasic activity are hypothesized to contribute to the emergence of compulsive drug-directed behavior and to the weakening of other motivated behaviors.

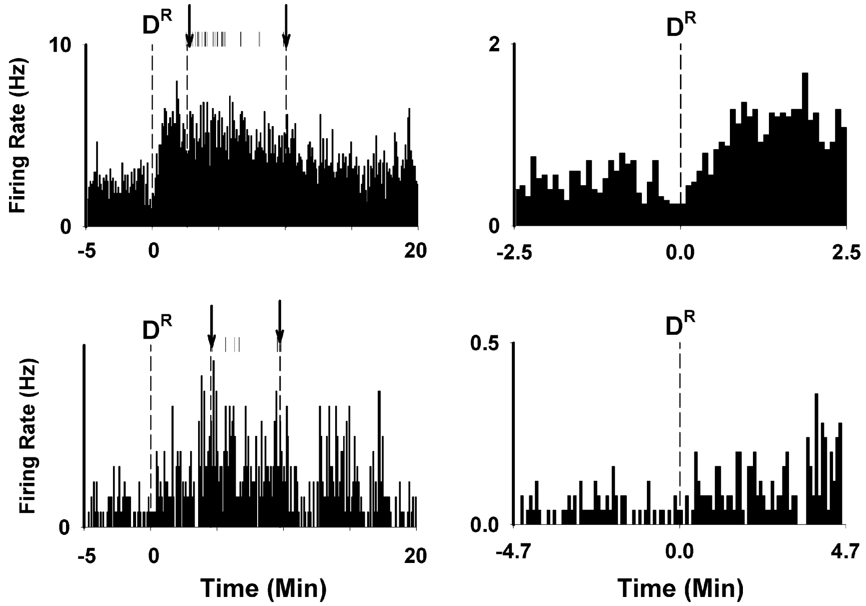

**FIGURE 4.** Examples of excitatory changes in firing during a cue + drug reinstatement procedure. Animals were exposed to a within-session extinction and drug + cue reinstatement procedure. This procedure was comparable to that described in Fig. 3 except that operant responding was reinstated by the combined presentation of a single infusion of cocaine (0.75 mg/kg/inf) and cues that were typically paired with the cocaine reinforcer. In this figure, firing rates before vs. after the onset of the drug + cue presentation are shown for each of two neurons. In all histograms, "$D^R$" marks onset of the drug + cue. (Left) Each histogram shows firing (discharges per sec, Hz) during pre- and post-$D^R$. The pre-$D^R$ period equals the 5-min period that immediately preceded the onset of the $D^R$. The post-$D^R$ period equals the interval that elapsed between the onset of the $D^R$ and 5 min after the last reinstated press. Small vertical bars above each histogram correspond to lever presses; first and last reinstated presses are marked with arrows. (Right) Each histogram displays firing during a pre- and post-$D^R$ period. The post-$D^R$ period corresponds to the interval that elapsed between the onset of the $D^R$ and the time of the first reinstated press. The pre-$D^R$ period is an equal interval that immediately preceded the onset of the $D^R$.

## Effects of DA and Cocaine on NAC Neural Activity

### *Acute Effects of DA on NAC Neural Activity*

There are at least five types of DA receptors expressed in the rat accumbens[98,99,100,101,102,103]. These receptors fit into two broad families, the D1-type receptors, which include the D1 and D5 receptor, and the D2-type receptors, which include the D2, D3, and D4 receptors. All of these receptors are G-protein coupled receptors that affect multiple ion channels and other effectors, and thus cannot be described as simply excitatory or inhibitory. Most evidence from NAC preparations indicates that D1 activation enhances glutamatergic transmission, whereas D2 activation is inhibitory[104,105,106,107,108,109, 110,111,112,113]. In this way, synapses that are receiving glutamate are facilitated and synapses that are not receiving glutamate are inhibited. These findings are consistent with the interpretation that DA effects on NAC neural activity are activity dependent.

An activity-dependent influence of DA on NAC activity is supported by recordings in awake animals. DeFrance and colleagues found that the effect of DA iontophoresis on NAC firing was dependent on the frequency of fimbria stimulation: DA inhibited NAC responses at 0.5 Hz stimulation, but not at 6.0 Hz. The authors concluded that, "the result of increased DA action could be viewed as an increase in the signal-to-noise ratio or a form of 'contrast enhancement'"[114](p. 1576). More recent work has been consistent with these findings. In rats, DA iontophoresis inhibited spontaneous striatal, but not glutamate-elicited, firing[115]. Based on these and other observations, it has been hypothesized that DA facilitates the transmission of strong excitatory input through the NAC, while simultaneously suppressing weak inputs[78,83,110,112,113,115,116,117].

## *Acute Cocaine Effects on NAC Neuronal Activity*

### Acute Experimenter-Delivered Cocaine Inhibits NAC Neuronal Activity

Acute electrophysiological recording studies generally show that experimenter-delivered cocaine inhibits spontaneous and glutamate-evoked firing of NAC neurons[118,119,120,121]. This inhibitory effect is mediated primarily by cocaine-induced elevations of accumbal DA and associated increases in activation of DA receptors (for review, see [104]; also see [4,115,120,122,123]). Evidence suggests that stimulation of the DA receptors initiates a cascade of cellular events that contribute to hypoactivity[124,125,126,127,128].

### Evidence that Acute Effect of Self-Administered Cocaine on NAC Neurons is Inhibitory

Extracellular recordings during intravenous cocaine self-administration (0.75 mg/kg/inf) sessions have shown that most (≈90%) NAC neurons exhibit a change in average firing rate during the self-administration session relative to the pre- and postsession baseline periods (referred to herein as session-long increases and decreases, Fig. 5)[67,68,129,130]. The session-long changes in firing are exhibited by neurons that exhibit phasic lever-press firing patterns, as well as neurons that do not exhibit phasic activity during the self-administration session. More than half of the session-long changes in firing are decreases. The session-long changes in firing are not attributable to specific motor behaviors (Fig. 6)[68]. We hypothesize that nonpharmacological changes in afferent input contribute to both the session-long increases and decreases, however, the session-long decreases in firing are additionally mediated by inhibitory pharmacological effects of cocaine.

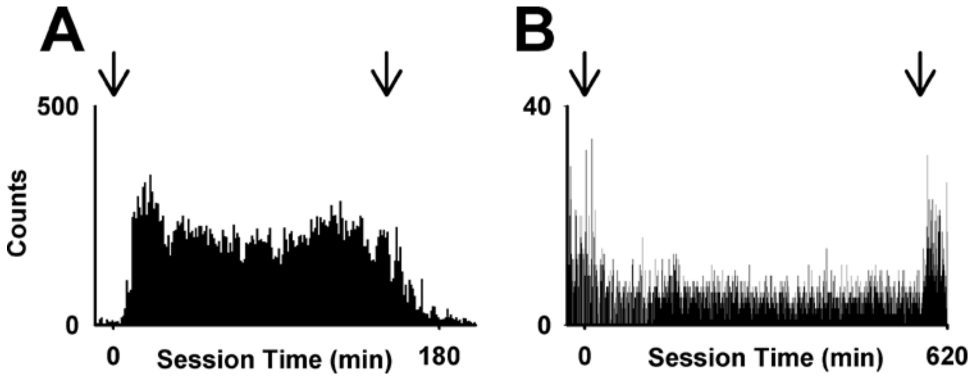

**FIGURE 5.** Examples of session-long firing patterns exhibited by individual neurons during a cocaine self-administration session. In each histogram, average firing rate (i.e., counts per 30-sec bin) of a single neuron is plotted as a function of time during the recording session. (A) An example of an increase in firing during the self-administration session relative to the presession drug-free baseline period and a postsession recovery period (i.e., session-long increase). Beginning and end of self-administration is indicated by arrows at top of histograms. (B) An example of a decrease in firing during the self-administration session (i.e., a session-long decrease).

The hypothesis is based on two observations made in behaving animals. The first of the observations is that session-long decreases show a tighter relationship to cocaine levels than do session-long increases. At the onset of the session, drug level increases progressively across the initial self-infusions. On average, session-long decreases, but not session-long increases, show a similar progressive onset (Figs. 6 and 7). These finding are consistent with the interpretation that drug actions potentially contribute to session-long decreases in firing, but not to session-long increases. Also consistent with this interpretation is the following. We recently observed session-long firing patterns during sessions in which animals self-administered sucrose. The prevalence of session-long increases was comparable between the sucrose and cocaine sessions (≈30%). The similar prevalences are consistent with the interpretation that session-long increases in firing reflect nonpharmacological mechanisms typically associated with reward-directed behavior. In contrast, the prevalence of session-long decreases in the sucrose study (≈30%) was half of that which we have observed during the cocaine self-administration studies (≈60%)[87]. It is possible that the increased number of session-long decrease neurons during the cocaine sessions reflects an inhibitory pharmacological effect of cocaine.

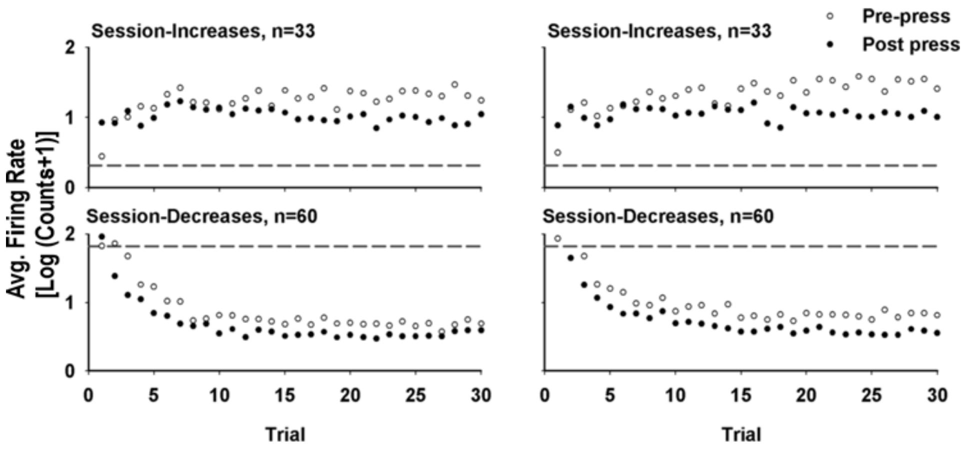

**FIGURE 6.** Session-long changes in firing are not attributable to specific motor behaviors. Average firing of all neurons identified as showing session-long increases (top two graphs) and decreases (bottom two graphs) during an FR1 cocaine self-administration session (0.75 mg/kg/inf). The figure shows firing of NAC neurons recorded in animals with a 2-week history of cocaine self-administration training (6 h/day)[68]. To construct this figure, all recorded neurons that showed either a session-long increase or a session-long decrease were identified. Average firing rates at various points during the self-administration session were then calculated for each of the two subsets of neurons. (Left column) Average firing rate [i.e., $Log_{10}$ (counts + 1)] during the 30 sec before and after each of the first 30 cocaine-reinforced lever presses of the self-administration session is plotted as a function of press (i.e., trial). Firing rates are plotted for all neurons that showed a session-long increase (top) and all neurons that showed a session-long decrease (bottom). The 30-sec prepress is a period of maximal drug-directed behavior (i.e., lever approach and lever pressing). The 30-sec postpress is an interval of minimal drug-directed behavior, during which animals locomote away from the lever. Firing during the two 30-sec intervals underwent similar changes across the session. (Right column) Average firing rates during the –40- to –10-sec prepress and the 10- to 40-sec postpress is plotted as a function of press number. Firing rates are plotted for all neurons that showed a session-long increase (top) and session-long decrease (bottom). The –40- to 0-sec prepress and the 10- to 40-sec postpress exclude the periods of time in which phasic firing patterns occur time locked to the lever press (i.e., –3 and +3 sec pre- and postpress). During both the –40- to –10-sec prepress period and the 10- to 40-sec postpress period, animals engage primarily in stereotypy. Firing during these intervals showed similar changes across the session. Moreover, the changes were similar to those observed when firing rates were calculated during intervals that included the period of the phasic lever-press firing patterns (shown in left column). Data shown in this figure are consistent with the interpretation that session-long changes in firing are not attributable to specific behaviors.

These observations are consistent with those that might be expected if the primary pharmacological effect of self-administered cocaine is inhibition. However, the findings are subject to alternative interpretations. There are numerous differences between cocaine- and sucrose-directed behaviors that could have contributed to the greater prevalence of session-long decreases in firing during the cocaine sessions relative to the sucrose sessions. Moreover, variations in dose and drug level over the course of the self-administration session alter variables, such as reward magnitude, that could possibly affect NAC activity,

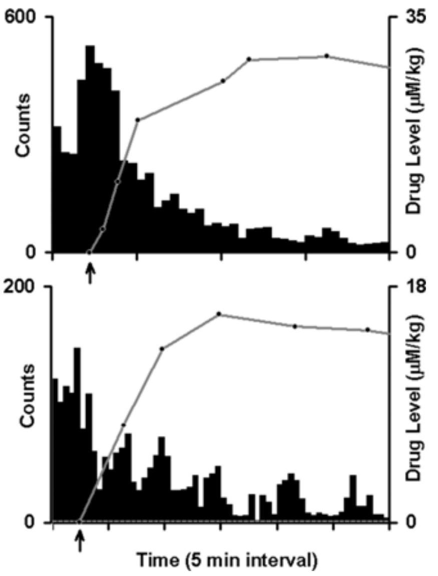

**FIGURE 7.** Example of a session-long decrease that developed progressively as drug level increased at the onset of a self-administration session. Average firing rate (Hz) is plotted as a function of 30-sec bins for a single neuron. Also plotted is calculated drug level[136]. Firing rate and drug level are shown for the first six self-infusions of the session. (Figure taken from Peoples et al. (2004) *J. Neurophysiol.* **91,** 314–323. With permission.)

independent of the primary effects of cocaine on NAC neurons. Additional studies are required to further characterize the pharmacological effects of self-administered cocaine.

## Relevance to the Differential Inhibition Hypothesis

Given the current understanding of DA and cocaine effects in the NAC, we have hypothesized that the acute pharmacological effects of self-administered cocaine are inhibitory; however, strongly activated neurons are less subject to the inhibitory effects of cocaine than are weakly activated neurons. The differential acute inhibition is expected to exaggerate differences in firing between neurons receiving strong excitatory afferent input and those receiving little or no excitatory input during the cocaine self-administration session. This amplification could correspondingly enhance the differential susceptibility of the neurons to activity-dependent plasticity mechanisms.

## Mechanisms that Mediate NAC Hypoactivity

Consideration of the mechanisms associated with NAC hypoactivity is consistent with the interpretation that those mechanisms could be activity dependent and, more specifically, opposed by excitatory afferent input. One activity-dependent mechanism linked to NAC hypoactivity is a decrease in the AMPA:NMDA current ratio[131], which is an LTD-like adaptation. The decrease in the current ratio associated with LTD is activity dependent. It is possible that the cocaine-induced changes are similarly activity dependent. Another mechanism that is thought to underlie cocaine-induced NAC hypoactivity is a decrease in $Na^+$ currents and an associated decrease in membrane excitability[3,132]. It is hypothesized that this effect of cocaine is mediated by (1) D1 receptor mechanisms that decrease release of intracellular $Ca^{2+}$ and (2) acute inhibitory effects of DA on firing rate of NAC neurons, which decreases $Ca^{2+}$ influx[120]. Evidence consistent with these mechanisms has been described[120]. These mechanisms may also be activity dependent. Specifically, increased excitatory activity is expected to increase $Ca^{2+}$

influx. An increased $Ca^{2+}$ influx would counter the DA-mediated decreases in intracellular $Ca^{2+}$ and thereby oppose the decreases in $NA^+$ currents that underlie the membrane hypoactivity.

### Relevance to the Differential Inhibition Hypothesis

Given the above observations, we have hypothesized that neurons that receive excitatory input during drug-directed behavior will be less susceptible than neurons that receive only weak excitatory input to cocaine-induced hypoactivity. Based on mechanisms that mediate other types of adaptations, the activated neurons might also be more susceptible to a number of excitatory neuroadaptations that have been observed to occur in association with repeated cocaine exposure[30,36,133,134].

### Summary of the Basis of the Differential Inhibition Hypothesis

The differential inhibition hypothesis is based on multiple observations related to the NAC. These findings provide evidence for the following: (1) specific spatial patterns of activity are associated with particular motivated behaviors, (2) acute DA effects on NAC neurons can be activity dependent, (3) the acute pharmacological effect of experimenter-delivered cocaine is inhibition, (4) the acute pharmacological effect of self-administered cocaine may also be inhibition, and (5) certain mechanisms that mediate hypoactivity are activity dependent.

## NOVEL PREDICTIONS OF THE DIFFERENTIAL INHIBITION HYPOTHESIS

Our laboratory has begun to test novel predictions of the differential inhibition hypothesis. Two predictions include the following. First, the cocaine-induced decrease in average NAC firing during cocaine self-administration sessions will be less for neurons that are activated during the sessions than for neurons that are not activated. For example, neurons that show an excitatory lever-press firing pattern are expected to maintain higher average firing rates during the self-administration session than are neurons that show no lever-press pattern. Second, the chronic inhibitory effect of repeated cocaine exposure will be less for neurons that are activated during cocaine self-administration sessions relative to neurons that are not activated during the sessions. For example, neurons that show excitatory lever-press firing patterns during cocaine self-administration sessions are expected to develop less hypoactivity across repeated sessions than are neurons that do not show a lever-press pattern. Available data are consistent with these predictions. Some of these data are described herein.

### Within- and Between-Session Comparisons of “Activated” and “Nonactivated” Neurons

In a recent study, we characterized the changes in firing exhibited by NAC neurons during individual self-administration sessions. In the same study, we also tested for between-session changes in activity. Rats were exposed to 30 daily long-access (6 h) cocaine self-administration sessions. Chronic extracellular recordings of the activity of individual NAC neurons were made during the $2^{nd}$–$3^{rd}$ and $30^{th}$ sessions of the regimen (i.e., referred to as early vs. late sessions). Each recording session included a presession baseline period, a cocaine self-administration session, and a postsession recovery period. Neurons recorded on each day were categorized into two groups, task activated and task nonactivated. Task-activated neurons were defined as (1) all neurons that showed a session-long increase in firing (e.g., Fig. 5A) and (2) all neurons that showed an excitatory response to a discrete task-related event, such as the lever-press operant or the cues that signaled the onset of the self-administration session (e.g., Figs. 1 and

2). All other neurons were defined as task-nonactivated neurons (see [67] for detailed methods). About half of all recorded neurons in both the early and the late session were task activated, while the other half were task nonactivated. The average firing of the two groups of neurons was compared both within each of the self-administration sessions and across the two recording sessions.

The within-session comparisons showed consistent differences between the average firing rates of activated and nonactivated neurons during cocaine self-administration sessions. During the early recording session, average firing rate of the task-nonactivated group significantly decreased relative to the presession baseline firing rate. In contrast, firing of the task-activated group did not significantly change during the self-administration session relative to the presession firing rate (Fig. 8). In association with the differential inhibition, average firing rate of the task-activated neurons remained significantly greater than firing of the task-nonactivated neurons throughout the self-administration session. The same differences between activated and nonactivated neurons were observed during the late recording session.

Comparisons between the early and the late session showed that the presession average firing rate of the task-nonactivated group was significantly depressed in the late session, relative to the early session. In contrast, the average presession firing rate of the task-activated group did not change between the early and the late session (Fig. 8). The differential between-session change in firing was associated with (1) the emergence of a significant difference in baseline firing rates of the activated and nonactivated neurons, (2) an increase in the ratio between the firing rates of the activated neurons and the nonactivated neurons, and (3) the occurrence of parallel changes in average firing during the cocaine self-administration and baseline recovery phases. The generality of the differential between-session change in firing across the phases of the recording session, and a number of other control analyses, ruled out multiple nonpharmacological explanations of the differential between-session changes, including learning.

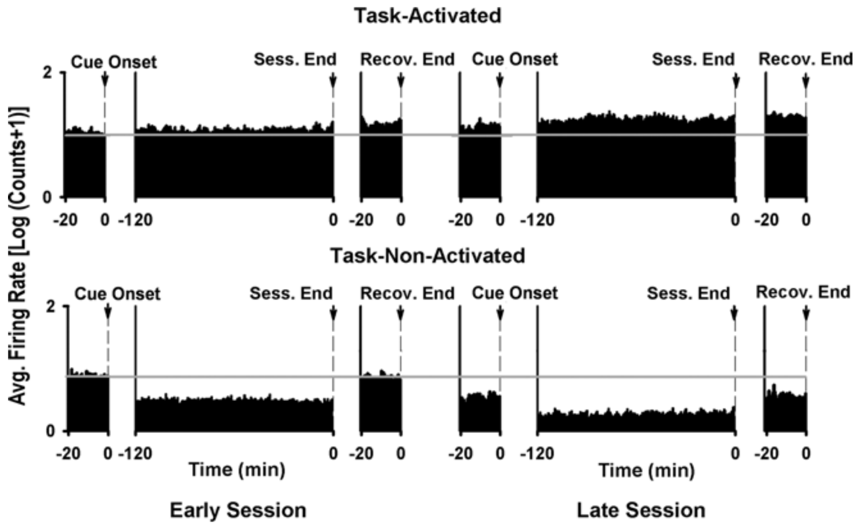

**FIGURE 8.** Between-session changes in average firing rate of task-activated and task-nonactivated neurons. (Top) Average firing of the task-activated neurons is shown during the early (left) and late (right) recording sessions. Firing rate is shown for the following periods: (1) the last 20 min of the presession baseline period, (2) the last 2 h of the self-administration session, and (3) the last 20 min of a baseline recovery period, which immediately followed the cocaine self-administration session. The solid gray horizontal lines correspond to average firing rates during the baseline phase of the early session. (Bottom) The same data are shown for the task-nonactivated neurons. (Figure taken from Peoples et al. (2007) *Neuropsychopharmacology* **32**, 1141–1158. With permission.)

In an additional analysis of the same data, we more finely sorted neurons based on their response characteristics. Specifically, neurons in the activated group were subdivided into two groups: (1) neurons that showed a session-long increase (referred to as session-increase neurons) and (2) neurons that showed a phasic increase in relation to a task event, but showed no session-long increase (referred to as task-but-not-session-activated). Replication of the original comparisons showed that the between-session change in firing exhibited by the three subtypes of neurons was positively related to the type of within-session change in firing exhibited by the neurons during individual cocaine self-administration sessions (Figs. 9 and 10). Session-increase neurons maintained elevated rates of firing throughout individual self-administration sessions and showed a trend to increase baseline firing rates between the early and the late sessions. Task-but-not-session-activated neurons maintained average firing rates during individual self-administration sessions that were similar to baseline firing rates and correspondingly showed no between-session change in baseline. Finally, task-nonactivated neurons were inhibited during self-administration sessions and showed a between-session decrease in basal activity. The findings are consistent with the interpretation that between-session changes in firing exhibited by neurons are directionally consistent with the change in firing exhibited by the neurons during individual cocaine self-administration sessions.

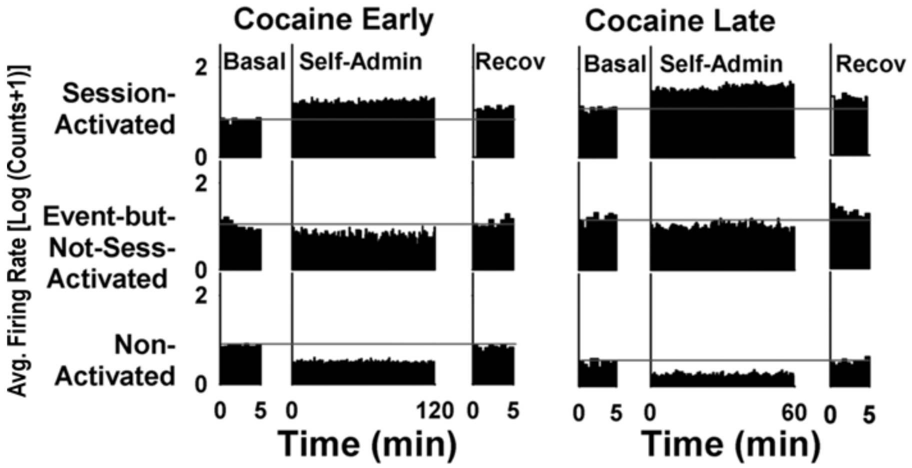

**FIGURE 9**. Group mean histograms showing average firing of groups of neurons during early and late cocaine self-administration sessions. Firing is shown during three periods in each condition: (1) the last 5 min of the presession baseline period, (2) the last 1–2 h of the self-administration session, and (3) the last 5 min of the postsession baseline (recovery) period. The histograms show average firing of the following three groups of neurons: (1) all session-increase neurons (top row), (2) all task-but-not-session-activated neurons (middle row), and (3) all nonactivated neurons (bottom row).

During the late session, differences between the basal firing rates of activated and nonactivated neurons were predictive of animals' drug-directed behavior during the later phases of the recording session. Animals with the greatest difference in basal firing between activated and nonactivated neurons consumed the greatest amount of cocaine during the self-administration session. The same animals also completed the highest number of lever-press responses during extinction and cue reinstatement probes that were conducted later during the recording session. The finding is suggestive of a relationship between the difference in basal firing between activated and nonactivated neurons, and the propensity of animals to engage in drug-directed behavior.

Overall, the data of this study are consistent with several predictions of the differential inhibition hypothesis, including the following. First, neurons receiving weak excitatory input in association with drug-directed behavior will show more depressed firing during the self-administration session relative to neurons receiving strong excitatory input. Second, neurons that do not receive strong excitatory input during cocaine self-administration sessions will exhibit evidence of hypoactivity (e.g., decreased basal activity) across repeated cocaine sessions. Moreover, neurons that receive strong excitatory input during cocaine self-administration sessions will not show evidence of the same inhibitory between-session change in activity

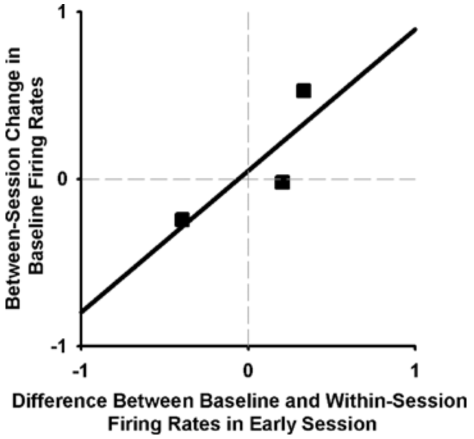

**FIGURE 10.** Relationship between the within-session and between-session changes in firing exhibited by three subtypes of neurons. Figure demonstrates that the between-session change in firing (ordinate) exhibited by three subtypes of neurons was directionally consistent with the type of *within*-session change in firing (abscissa) exhibited by the neurons. Neurons that showed an increase, no change, and a decrease in firing during individual cocaine self-administration sessions showed a corresponding *between*-session increase, no change, and decrease in firing. The three neuron subtypes include the following: (1) all session-increase neurons (top right point on line), (2) all task-but-not-session-activated neurons (midpoint on line), and (3) all nonactivated neurons (bottom left on line). For each subtype of neuron, the measure of between-session change corresponds to the difference in average presession baseline firing between the early recording session (i.e., days 2–3 of the long-access cocaine self-administration regimen) and the late recording session (i.e., the 30$^{th}$ day of the regimen). For each subtype of neuron, the measure of within-session change in firing equals the difference in firing between the last 1 h of the self-administration session and the 20-min presession baseline period.

and may, in fact, show evidence of excitatory between-session changes in firing. Third, the differential between-session changes in firing will shift basal activity of neurons so that it is more consistent with that which is observed during cocaine self-administration sessions and amplify the difference in firing between activated and nonactivated neurons during drug-directed behavior. Fourth, the differential between-session changes in firing will be associated with an increase in drug-directed behavior.

## Additional Predictions: Changes in Phasic Firing Patterns Associated with Drug-Directed Behavior

The study described above shows that groups of neurons that are differentially activated in relation to drug-directed behavior respond differently during individual sessions, as well as across repeated drug sessions. These findings provide "between-neuron" evidence of activity-dependent acute and chronic effects of cocaine on NAC neurons. Based on previous studies of DA, we hypothesized that we might also observe within-neuron evidence of similar activity-dependent effects of cocaine. In the DA studies, iontophoretic application of DA inhibited the activity of individual neurons, however, the inhibition of the same neurons was reduced or absent if DA was applied concurrently with glutamate. Given these findings, we anticipated that neurons that were phasically active during the cocaine self-administration session would show a related activity-dependent response to cocaine. For example, firing of a lever-press activated neuron might be maintained or even enhanced during the period of a cocaine-reinforced lever press (i.e., during the signal period) relative to the periods of the self-administration session in which the lever-press event was absent (Fig. 11). Moreover, these differential within-session changes in signal and background firing were expected to be associated with parallel between-session changes in signal and background firing.

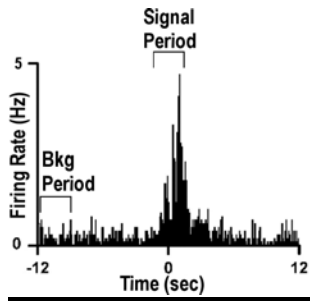

**FIGURE 11.** Signal and background periods. Shown are the signal and background periods for a neuron that exhibited a phasic increase in firing time locked to the cocaine-reinforced lever press (referred to as a lever-press pattern). The histogram shows average firing rate (i.e., discharges per sec, Hz) per 0.1-sec bin during the 12-sec pre- and postpress. Brackets indicate signal and background (Bkg) periods. (Figure taken from Peoples and Cavanaugh (2003) *J. Neurophysiol.* **90**, 993–1010. With permission.)

Available evidence is consistent with the first prediction. In one study, we observed evidence of differential changes in signal and background firing and increased signal:background ratios of excitatory lever-press firing patterns within individual self-administration sessions[66]. Published reports relevant to the second prediction are mixed. However, in the Peoples et al. experiment described above[67], comparisons between the early and late sessions did not reveal any evidence of between-session change in either the prevalence or amplitude of lever-press firing patterns. However, Hollander and Carelli[39] found that in animals with a history of repeated cocaine self-administration sessions, the prevalence and amplitude of phasic lever-press firing patterns was increased at a late abstinence time point relative to an early abstinence time point. The increased amplitude of the excitatory lever-press firing patterns was associated with an increase in average signal firing. One potential explanation of the between-experiment differences in results is that adaptations that differentially influence signal and background firing occur across repeated cocaine self-administration sessions, but the impact of those adaptations on NAC activity differ between early and late abstinence periods. The effects of repeated cocaine self-administration on phasic firing patterns require further study. However, the findings of the Peoples et al.[67] and Hollander and Carelli studies[39,40] are consistent with the prediction that activated neurons will either maintain or increase activity rather than show evidence of hypoactivity.

## SUMMARY AND FUTURE RESEARCH DIRECTIONS

Multiple lines of evidence support the conclusion that repeated cocaine exposure leads to hypoactivity in the NAC. The hypoactivity has been proposed to contribute to cocaine addiction. There are two important questions related to this hypothesis. First, various types of investigations have documented neurochemical and molecular adaptations that could underlie NAC hypoactivity. However, there is also evidence of other adaptations in the NAC, and in NAC afferents, which are expected to have an excitatory influence on NAC neural activity. The evidence for excitatory as well as inhibitory adaptations raises questions as to whether and how NAC hypoactivity contributes to cocaine addiction. Second, cocaine addiction is characterized by an increase in drug-directed behavior and a simultaneous weakening of other motivated behaviors. However, the NAC contributes to both drug- and nondrug-motivated behavior. Moreover, the nature of the contributions is similar and associated predominantly with excitatory phasic firing patterns. Given these observations- it is not clear how hypoactivity of NAC neurons might contribute to the behaviors that characterize cocaine addiction.

We hypothesize that drug-directed behavior is associated with a particular spatiotemporal pattern of NAC afferent input: neurons receive strong excitatory input whereas others do not. When drug-directed behavior leads to cocaine exposure, the acute pharmacological actions of cocaine inhibit NAC neurons in an activity-dependent manner. The differences in afferent input and acute effects of cocaine contribute to differential neuroplasticity. The differential neuroadaptations are hypothesized to alter the basal spatial pattern of synaptic and neuronal excitability and activity so that it is chronically more like that which is present during cocaine self-administration. This may chronically facilitate drug-directed behavior, while simultaneously decreasing the expression of other motivated behaviors.

Our initial tests of the hypothesis have been positive. For example, task-nonactivated neurons are more inhibited than are task-activated neurons within individual self-administration sessions. Correspondingly, task-nonactivated neurons show between-session decreases in basal firing rates, whereas task-activated neurons show either no change or an increase in firing. More work is necessary to fully characterize the pharmacological nature of these differential changes in NAC firing. There are also a number of predictions of the differential inhibition hypothesis that we have yet to test. Independent of the outcome of the additional investigations, further study of the differential inhibition hypothesis could facilitate progress in understanding whether and how drug-induced NAC hypoactivity contributes to cocaine addiction. Moreover, further studies might help to integrate physiological studies of the NAC with other investigations of cocaine-induced neuroadaptations, which will be important to developing effective therapeutic interventions.

## ACKNOWLEDGMENTS

We thank Alexis Simpson for expert technical assistance. LLP and AS supported by NIH/NIDA P60 DA 005186 (CP O'Brien), NIH/NIDA P50 DA 012756 (H Pettinati). KG supported by NIH Director's Bench-to-Bedside (LLP). AVK supported by DA-07241(CP O'Brien) and DA-021449 (AVK). Authors would also like to thank Dr. Susan Volman for her helpful comments on an earlier version of the review.
